# Practical considerations in the establishment of psychedelic research programs

**DOI:** 10.1007/s00213-024-06722-6

**Published:** 2024-12-04

**Authors:** Brian S. Barnett, M. Frances Vest, Marcus S. Delatte, Franklin King IV, Erin E. Mauney, Anthony J. Coulson, Sandeep M. Nayak, Peter S. Hendricks, George R. Greer, Kevin S. Murnane

**Affiliations:** 1 Department of Psychiatry and Psychology, Neurological Institute, Cleveland Clinic, Lutheran Hospital, 1730 W 25th Street Floor 5E, Cleveland, OH 44113 USA; 2 Cleveland Clinic Lerner College of Medicine at Case Western Reserve University, EC-10 Cleveland Clinic, Cleveland, OH 44195 USA; 3 Department of Pharmacology, Toxicology and Neuroscience, Louisiana State University Health Sciences Center, Shreveport, LA 71130 USA; 4 Regulatory and Drug Development Consulting, Allucent, Cary, NC 27513 USA; 5 Department of Psychiatry, Center for Neuroscience of Psychedelics, Massachusetts General Hospital, Boston, MA 02114 USA; 6 Division of Gastroenterology and Nutrition, MassGeneral Hospital for Children, Boston, MA 02114 USA; 7 NTH Consulting, Inc, Tucson, AZ 85715 USA; 8 Center for Psychedelic and Consciousness Research, Johns Hopkins University School of Medicine, Baltimore, MD USA; 9 Department of Psychiatry and Behavioral Neurobiology, School of Medicine, University of Alabama at Birmingham, Birmingham, AL 35294 USA; 10 Heffter Research Institute, 15 E Putnam Ave, Ste 413, Greenwich, CT 06830 USA; 11 Department of Psychiatry, Louisiana State University Health Sciences Center, Shreveport, LA 71130 USA

**Keywords:** Clinical trials, Psychedelics, Psilocybin, LSD, Clinical research

## Abstract

**Rationale:**

There is increasing interest in establishing psychedelic research programs at academic medical centers. However, psychedelics are intensely psychoactive, carry considerable sociopolitical baggage, and most are Schedule I drugs, creating significant potential impediments to implementation. There is little formal guidance for investigators on navigating the complex on-the-ground obstacles associated with establishing psychedelic research programs.

**Objectives:**

This article provides recommendations that may be helpful to investigators seeking to work with psychedelics, with a focus on academic medical centers in the United States.

**Methods:**

The academic literature on relevant matters is reviewed, and the authors provide observations from their experiences either working for relevant regulatory agencies or conducting basic science studies, investigator-initiated trials, or industry sponsored trials with psychedelics.

**Results:**

Investigators planning to conduct psychedelic research should cultivate broad institutional support early. Challenges related to securing funding, obtaining approval for an Investigational New Drug application from the Food and Drug Administration, clinical grade drug sourcing, obtaining a Schedule I researcher registration from the Drug Enforcement Administration and an equivalent state license (if required), preparing spaces for treatment and study drug storage, managing controlled substance inventory, engaging the local community, and other issues should be anticipated.

**Conclusions:**

Investigators should anticipate several implementation challenges when planning to work with psychedelics. However, these are likely surmountable with planning, persistence, and assistance from colleagues and other experts.

## Introduction

Amid steadily growing interest in the therapeutic potential of psychedelics, as well as increasing industry (Aday et al. 2023), state (Acquisto and Six 2023), and federal (Volkow et al. 2023) financial support for this area of research, many academic medical centers throughout the United States have recently begun conducting studies of psychedelics or are planning to do so in the near future (Basen 2021). However, the intense psychoactive effects of these compounds, their Schedule I designation under the Controlled Substances Act (United States Code of Federal Regulations 2024a), and their historical notoriety present unique challenges to conducting research with psychedelics. While the United States Food and Drug Administration (FDA) has released guidance on psychedelic clinical trial design and personnel qualifications (United States Department of Health and Human Services 2023), there is little formal instruction for investigators on how to navigate the complex, on-the-ground obstacles frequently associated with erecting the infrastructure necessary for conducting research with psychedelics. Therefore, the aim of this article is to distill the experience of the authors, who have conducted basic science studies, investigator-initiated trials (IITs), or industry sponsored trials with psychedelics or have been previously employed by the FDA (author MSD) or the United States Drug Enforcement Administration (DEA) (author AJC), into a guide that may be helpful to investigators and administrators seeking to establish psychedelic research programs, particularly in academic medical centers in the United States.

### Cultivating institutional support

Sustainable psychedelic research programs should be designed to successfully leverage regulatory, scientific, and clinical expertise and infrastructure. While rewarding, establishing such programs can be a demanding and frustrating endeavor, particularly when viewed in comparison to the execution of traditional mental health-related clinical trials of behavioral interventions and daily medications. Due to regulatory structures, it can take one to two years–or longer–after a study protocol is developed before the first dose of a psychedelic is administered to a participant. Therefore, planning and early engagement with stakeholders are essential to minimizing delays.

Having an advanced understanding of which departments and offices in your organization will need to be involved in your efforts and providing ample notice to them about your intentions can help prevent setbacks to study launch. Due to the complexities of psychedelic research, input is often required from a broader team of colleagues in an organization than principal investigators might initially anticipate. Depending on the type of research you plan to conduct, you may need to collaborate with colleagues in some or all the departments listed in Table 1, as well as external collaborators (discussed below). In this regard, there is often heterogeneity in research and clinical infrastructure and expertise between departments within an academic medical center, which can be exacerbated when those departments are divided explicitly into colleges or other structures that separate scientific and clinical disciplines.

**Table 1:** Departments frequently involved in implementing psychedelic research programs

Department	Role in psychedelic research
Billing and compliance	Ensuring clinical trials are conducted in accordance with regulatory requirements, including management of controlled substances and the correct application of billing codes and reimbursement processes
Clinical engineering	Maintenance of study equipment such as vital sign machines and electrocardiogram machines
Environmental services	Cleaning of treatment room and laundering of bedding
Information technology/Cybersecurity	Developing electronic medical record treatment delivery “context” for research area and determining whether treatment sessions can be recorded in a compliant manner or if participants can use phone applications to record data
Institutional animal care and use committee	Determining whether a study protocol involving non-human vertebrate animals will be approved
Institutional review board	Determining whether a study protocol involving humans will be approved and allowable methods for recruitment
Laboratory	Processing and/or storage of participant laboratory specimens
Legal	Contract negotiations and informed consent review
Pharmacy	May or may not directly manage investigational product; will conduct quality assurance reviews of drug storage
Point of care testing	Determining which point of care tests can be used
Receiving	Receiving study related parcels; investigational product may be delivered here
Research nursing	Conducting study visits, obtaining laboratory specimens, and administering study drug to participants, particularly when parenteral administration is required
Security	Providing access (key/swipe card) controls to staff for study spaces and managing security camera footage
Space committee	Allocating space for treatment and investigational product storage
Regulatory Affairs	Supporting the development of regulatory strategies and documents for the FDA and other related groups

Despite frequent media coverage of advancements in psychedelic medicine, many individuals outside of psychiatry, psychology, and neuroscience (and even within these fields) remain unaware that psychedelics are being investigated for clinical applications. It can be helpful to provide information about psychedelic research to colleagues whose assistance you may require, either via discussion or by sending copies of recent relevant lay or scientific publications. Laying this groundwork is especially key when working on multidisciplinary studies involving specialists from fields outside of psychiatry and psychology. Creating a psychedelic research interest group in your institution may be particularly helpful in exposing colleagues to psychedelic science, generating potential collaborations, and increasing institutional buy-in. We emphasize here the importance of remaining dispassionate and objective in the study of psychedelics, avoiding the quasi-religious stance that has come to characterize some elements of the “psychedelic movement” (Evans 2023).

We recommend conferring with your department chair before moving forward with any plans to launch a psychedelic investigation. This is important for gaining broader institutional buy-in and/or approval for your work and may also be necessary to secure funding for aspects of research infrastructure that may not be covered by external funders or may need to be in place to convince external funders of study feasibility, such as spaces for treatment and secure storage of study drug. Likewise, it will be important to consult with research leadership at your institution to obtain support beyond your own department. The Director of Research, Director of Clinical Research, and/or the Vice-Chancellor for Research (or similar institutional official) are key stakeholders whose support you will need to navigate the complexities of psychedelic research in a major academic institution. Academic medical centers operationalize clinical research through laboratory/department-dedicated, centralized, or hybrid personnel and resources. The key leader of centralized resources will take on additional importance depending on setting. The initial approach to these leaders and administrators may be rendered more effective if the researcher comes prepared to deliver a brief educational session on the current evidence for therapeutic applications of psychedelics, to assuage any concerns and preempt stigma-driven responses.

Additionally, numerous institutions have Regulatory Affairs offices to support research programs with commercial interests. These offices may assist in the development of strategies to engage with regulatory authorities and preparation of documents for supporting product development programs. In the absence of these offices, external collaborators (e.g., regulatory consultants) may be contracted to support research programs with commercial interests. Research pharmacy staff should also be involved early (see “Collaboration with pharmacy” section below).

Importantly, academic medical centers are often central to the cultural, economic, medical, and reputational standing of a community, particularly when situated in small or mid-size cities. As such, many members of the local community have important psychological and tangible interests in the success of their local academic medical center. Many of these individuals represent populations that are likely to be engaged in clinical research related to psychedelics and can offer important insights on cultural competency and community engagement. These people and other key community leaders should be consulted.

### Acquiring funding

While opportunities to participate in multisite industry sponsored clinical trials of psychedelics are rapidly increasing for clinical researchers amid efforts to commercialize psychedelics (Aday et al. 2023), securing funding arguably remains the most significant challenge to conducting investigator-initiated psychedelic research. Until recent years, studies on therapeutic applications of psychedelics were primarily funded by non-profit organizations with a focus on advancing psychedelic research including Heffter Research Institute, the Multidisciplinary Association for Psychedelic Studies (MAPS), and RiverStyx Foundation, as well as wealthy philanthropists. Unfortunately, some commentators have observed that obtaining philanthropic support for psychedelic research has unexpectedly become more difficult in recent years due to commercialization efforts leading potential philanthropists to wonder, “Why would I donate when I can invest? (Powell 2021; Psychedelic Alpha 2024)”.

Governmental funding for therapeutic psychedelic research is limited, though increasing. The United States National Institutes of Health (NIH) did not begin directly supporting contemporary research investigating therapeutic applications of psychedelics until the National Institute of Mental Health (NIMH) funded a career development grant to explore psilocybin’s mechanism of action in the treatment of Obsessive-Compulsive Disorder (OCD) in April 2021 (National Institutes of Health 2021a; Barnett et al. 2022). Soon after, the National Institute on Drug Abuse (NIDA) funded a multisite trial of psilocybin-assisted therapy for tobacco use disorder in September 2021 (National Institutes of Health 2021b). Since then, NIMH has issued guidance for grant applicants seeking to study therapeutic applications of psychedelics in human and animal studies (National Institutes of Health 2022), while NIDA has issued multiple requests for applications (RFAs) related to investigating psychedelics as treatments for substance use disorders (National Institutes of Health 2023a, b, c). The Department of Veterans Affairs (Department of Veterans Affairs 2024) and Department of Defense (Jaeger 2023) have also begun supporting psychedelic clinical research, as have the states of Arizona and Texas (Ernst 2021; Osmonbekov 2023). Multiple other states are considering funding proposals of their own, though a high profile attempt to dedicate a portion of Kentucky’s opioid-settlement money to studying ibogaine recently foundered (Watkins 2024).

While the number of organizations offering financial support for psychedelic research is increasing, the dramatic growth of interest in psychedelics within the scientific community means securing funding remains a highly competitive endeavor. Therefore, investigators should thoroughly explore any opportunities for internal institutional support and consider contacting their institution’s philanthropy division. High profile giving for psychedelic research has often come from wealthy alumni invested in their alma matter taking up this work (Johns Hopkins Center for Psychedelic and Consciousness Research 2024) or philanthropists with geographic ties to a particular institution (Wisconsin Health News 2021). It is worth noting that many investigators who have successfully secured philanthropic support for psychedelic research have benefited from a confluence of serendipity and effective personality traits that have crucially aided fundraising efforts in an otherwise highly competitive and challenging environment. Investigators seeking to attract external support may benefit from recognizing the importance of these non-scientific factors, since relying on being a skilled researcher alone could be insufficient for achieving this goal. Importantly, when talking with potential donors, it is essential for the field’s integrity to avoid making claims of therapeutic benefits from psychedelics not supported by data, while acknowledging the complexities and expenses of conducting psychedelic studies that might generate such data.

### Core study staff

In general, research programs designed to develop therapeutic products have a core staff that includes nurses, medical specialists (e.g., psychiatrists, cardiologists), phlebotomists, and research assistants. Although the roles for these and other staff may vary depending on study needs, all staff should have adequate training to ensure participant safety and that the planned study can be conducted according to the approved protocol. The FDA, institutional review board (IRB), and other regulatory agencies will evaluate the adequacy of key staff member qualifications when determining if a clinical protocol may proceed under an investigational new drug (IND) application. Study staff should be properly vetted to support human studies to avoid the FDA issuing a “clinical hold,” (Goldstein 2023) which would prevent study initiation. If the clinical trial aims to employ “psychedelic-assisted therapy,” the FDA may require additional qualifications for study therapists, such as having at least one licensed therapist (assuming a dyadic model) with a graduate training credential (MD, PhD, MSW, etc.), as specified in recent guidance (United States Department of Health and Human Services 2023).”

### Collaboration with pharmacy

When planning to administer psychedelics to humans, strong collaboration with your institutional pharmacy is essential to success. Most academic medical centers have an investigational drug service and dedicated research pharmacies to receive, manage, and securely store study drug. However, investigators may need to manage their own study drug if their research pharmacy is unwilling to manage Schedule I drugs or the study area is offsite. In some instances, the collaborating pharmacist may be required to obtain permission or licensing from the state’s Board of Pharmacy, or appropriate regulatory authority, to manage Schedule I drugs intended for research. Thus, even if the pharmacist is willing, they may not be allowed to store and manage the study drug. However, if not managing study drug, an institutional pharmacy can still provide recommendations on safe handling, secure storage, and inventory controls/protocols.

You should determine early on whether your pharmacy is able to assist in preparing study drug for weight-based studies or studies requiring non-oral routes of administration (i.e., intravenous). If these services are necessary and not offered by your pharmacy, it can vastly complicate matters and possibly prevent study execution.

You must prepare a standard operating procedure (SOP) in conjunction with your pharmacy that outlines details related to delivery of study drug to the facility, access controls, staff with access to study drug and study drug storage room, study drug management procedures (such as temperature monitoring and drug count reconciliation), how and where logs and other documentation are stored (both during a study and after completion), transportation of drug to pharmacy if necessary (i.e. for preparation), administration of study drug, sequestration of drug, and destruction of study drug or return to distributor/sponsor. The DEA will require this at the time of application (DEA 225 submission, new drug code, or protocol amendment). The local DEA Diversion Investigator will review this with you at the on-site inspection. States with controlled substance boards will also require an SOP for study drug management and storage.

It is important to understand what requirements your organization has for controlled substances since your Schedule I researcher registration makes you professionally and personally responsible for an accurate and timely inventory. Oversight and accountability will vary from institution to institution. For example, some institutions may require witnessed daily counts of controlled substances, while others require less frequent counts. Though pharmacy staff may not be managing study drug, they will still be responsible for conducting quality assurance reviews, so you must determine the frequency of those and what they require of you. The DEA only requires, based on the Code of Federal Regulations (CFR), that the inventory be conducted biennially (United States Code of Federal Regulations 2024b). A best practice is to conduct an inventory at least once per month and document this in your inventory log.

### Key external collaborators

Key external collaborators may vary depending on the expertise of the core study staff and others at your institution. External collaborators may include experts in areas such as toxicology, biostatistics, chemistry manufacturing and controls, regulations, and project management. These experts typically assist in developing and implementing strategies that involve their discipline at different stages of the program and may also be tasked with identifying risks in the program and recommending approaches to mitigate them. Experts may be academic faculty, consultants, or other professionals with relevant skills and experiences. The selection of key collaborators should be based on how well the skill and experience levels of each matches the program needs and budget. Of note, there are companies/consultants specializing in the training of study therapists in the delivery of psychedelic-assisted therapy, preparation of IND applications, and working with the DEA, who can assist you in these areas.

### Securing and preparing study spaces

Securing and preparing spaces for psychedelic administration to human participants and study drug storage can be challenging given the unique features needed to ensure adequate participant safety and experimental control and prevent drug diversion. Space is often in high demand at academic medical centers and there can be long delays in gaining approvals from space committees for space assignment and facility management for required alterations. The process to secure, renovate, and/or alter these spaces can take several months to possibly a year or longer. If you are interested in conducting either industry-sponsored trials or IITs, you should secure and prepare necessary spaces at the front end of the process, as they will be an integral component of feasibility assessments conducted by sponsors/funders evaluating potential trial sites and essential requirements for federal, state, and institutional regulators.

We recommend treatment rooms be in quiet areas, preferably away from building exits or stairwells. The importance of setting’s impact on psychedelic effects in humans has long been promoted (Hartogsohn 2017), and guidelines for psychedelic research with human participants state that “an aesthetically pleasing environment may decrease the probability of acute psychological distress (Johnson et al. 2008).” Therefore, treatment rooms are typically designed to resemble living rooms, with a couch or bed that participants can lie on, soothing artwork, and comfortable chairs for session monitors. The presence or appearance of medical equipment should be minimized to the greatest extent possible. Since rooms in medical centers often have fluorescent lighting, which is bright and can make audible sound, we recommend replacing this with other types of lighting such as incandescent or LED lamps. Due to fall risks when consciousness is altered, an ensuite restroom or a restroom close to the treatment room without the need to be in contact with others is preferred. Study staff should either have a key to the restroom or the locking feature should be removed from the door for safety. Additionally, bathroom mirrors should also be removed due to the risk of participant harm if broken. Treatment room windows should be locked and preferably composed of shatter resistant material. Artwork hardware should also be constructed of shatter resistant material, such as plexiglass. Many hospital rooms have speakers or flashing alarms in the ceiling that should be removed. Treatment rooms should also have strong Wi-Fi access to play session music playlists and allow for data collection from electronic devices if applicable.

Federal regulations for storing Schedule I substances vary according to whether an investigator is a practitioner (a physician or other health care professional licensed under state law) or a non-practitioner (a researcher who conducts animal or laboratory research and does not have a practitioner’s license). According to the Code of Federal Regulations (United States Code of Federal Regulations 2024c), practitioners must store Schedule I substances “in a securely locked, substantially constructed cabinet” (United States Code of Federal Regulations 2024d). In some cases, the DEA and some state regulatory agencies may deem a medication-dispensing system, such as a Pyxis™, already present in the research pharmacy sufficiently secure. Storage requirements are more complex for non-practitioners, and interested readers should refer to 21 Code of Federal Regulations (CFR) § 1301.72 (United States Code of Federal Regulations 2024e) for specific guidance.

*The following recommendations are predicated on the fact that the local DEA Diversion Investigators’ framework for distance from storage to dosing and other factors can vary. It is incumbent upon the Principal Investigator to advise the local DEA Diversion Investigator about the spatial relationship between the study drug storage space and treatment space prior to the site inspection.* We strongly recommend selecting a secure study drug storage space that is as close as possible to the site of administration. Transporting study drug over long distances within a building to the site of administration or preparation area (i.e., pharmacy) may prove complicated, requiring additional chain of custody documentation and sometimes escorts by facility police during transportation. More complicated still is transferring study drug from the location of the address of the DEA Schedule I researcher registration to an external site for administration, which may also require a police escort. In this case the study drug must be directly taken to the administration area upon arrival at the non-registered address and under no circumstances can it be removed and stored, even temporarily, at the non-registered address (even if only briefly locked in a room or safe). These situations must be fully discussed with the local Diversion Investigator during the planning process and prior to any administration of study drug to a participant. When considering whether to allow off-site dosing the DEA will consider factors such as local crime rates and distance to local police departments. Importantly, we are aware of sites where such permission was not granted.

Due to potential adverse acute psychological effects from psychedelics, it is advisable (and may be required by your facility) to have rescue medications near or in the treatment area. This may need to be stored in a Pyxis™ machine, which will require electrical and ethernet connections. Additionally, since controlled substances such as benzodiazepines may be kept on hand, pharmacy requirements may dictate that the Pyxis™ have back up electricity access, which may not be available in older buildings. Since a Pyxis™ can be disruptive to the aesthetic of the treatment room, we recommend placement in the secure study drug storage room or another area close to the treatment room.

Some facilities may also require that an automated external defibrillator (AED) or crash cart be near the treatment area and/or that a Medical Emergency Team (MET) or an Advanced Cardiovascular Life Support (ACLS) trained physician be on site, though there is considerable variation in these requirements. The authors are unaware of any psychedelic clinical trials where an AED, basic life support (BLS), MET, or ACLS have needed to be used.

### Electronic medical record (EMR) incorporation of studies

It may be necessary to have a new treatment area context created in the EMR to allow for scheduling of treatment sessions and billing. EMR orders for study drug will also likely need to be created. The study team may also want to have order sets constructed for easy ordering of as-needed medications. If your organization has an investigational drug service or research pharmacy, they may be able to help navigate the creation of these items more quickly.

### Laboratory specimen processing and storage

In determining study feasibility, it is important to assess the processing and storage needs for laboratory specimens. For example, some specimens require the use of a centrifuge or a refrigerated centrifuge before shipping. If you do not have these in your research area, it may be necessary to use those in your facility’s clinical laboratory if permitted to do so. Samples may need to be stored in freezers with different temperature capabilities if they cannot be shipped to outside laboratories same day. Therefore, determine whether your clinical laboratory has such freezers onsite or if they are available at a satellite location via courier. You should also have adequate storage for unused laboratory kits for clinical trials.

### Point of care testing approval

The FDA as well as IRBs may require obtaining urine or breath alcohol testing, urine drug screens, and urine pregnancy tests (if applicable) on treatment session mornings prior to dosing in human psychedelic studies. Quick turnaround time is needed for results, which necessitates use of point of care testing. Some organizations have strict guidelines around which point of care tests researchers can use, and if sponsors of industry trials are requesting use of a non-approved brand, approval of these tests from your organization’s point of care testing division may be necessary. You may also need to obtain a Clinical Laboratory Improvement Amendments (CLIA) certification, or your samples may need to be tested in duplicate, using the sponsor’s point of care test and your organization’s standard point of care test.

### Study equipment

Human psychedelic studies require equipment such as vital sign machines, electrocardiogram (ECG) machines, and ideally, audio players with the capability of broadcasting to headphones while playing on a speaker simultaneously to allow for continuous music exposure, assuming a program of music will be used during acute psychedelic effects. Clinical engineering may need to routinely check that vital sign machines, ECG machines, and other clinical equipment are functioning appropriately, and sponsors may request logs of these checks. We advise ordering any equipment, as well as treatment room furniture, several months in advance of study initiation in case of supply chain issues. Regarding ECGs, it is also important to consider whether study staff can interpret these or whether an arrangement will need to be made with clinicians in internal medicine or cardiology. Depending on what is available in your institution, you may also need phlebotomy and laboratory capabilities (i.e., a centrifuge) in your research area for collection and processing of blood samples.

### Study design considerations

In designing human studies, we recommend consulting the non-binding recommendations for the design of psychedelic clinical trials prepared by the FDA (United States Department of Health and Human Services 2023). These are aimed both at industry- and academic-sponsored trials. They discuss material to be included in IND applications, as well as adverse event reporting, efforts to minimize bias, who qualifies to serve as a treatment session monitor, and other important matters. Adverse event reporting should be given special consideration since it can be challenging to determine which aspects of the psychedelic experience qualify as adverse events.

The design of human studies is typically informed by peer-reviewed publications containing clinical and nonclinical data, information from data sources maintained by US regulatory agencies and related organizations, and original studies conducted by the sponsor. Published data and data from original studies completed by the sponsor can be leveraged to support planned human studies that mimic them, as closely as possible, in terms of dose schedule, patient population, and other key study design features. Data sources maintained by US regulatory agencies may also be used in the same manner. For example, the inactive ingredient database maintained by the FDA is typically leveraged to support the safety of drug product excipients administered in planned human studies using the same route of administration and maximum daily levels equal to or less than that deemed safe by the FDA (United States Food and Drug Administration 2023). Across sources, the FDA will evaluate the adequacy of the aforementioned data based on the quality of the design of the studies that collected them.

### Informed consent for clinical trials

Informed consent documents for psychedelic clinical trials should discuss potential adverse effects and approaches to manage them. These forms typically discuss common acute drug effects and important risks including challenging experiences, anxiety/panic, paranoia, heightened emotional reactivity, suicidality, psychosis, hallucinogen persisting perception disorder, serotonin syndrome, tachycardia, high blood pressure, headaches, seizures, and reproductive risks, as well as unknown risks. On account of the sometimes profound nature of the psychedelic experience and the possibility of enduring changes in personality (Bouso et al. 2018) and worldview following psychedelic exposure, informed consent in psychedelic clinical trials is an area of growing interest (Jacobs 2023; Barber and Dike 2023; Seybert et al. 2023). Due to limited data, it can be challenging to articulate what the actual risks to participants might be in these regards. However, the possibility of these changes does warrant inclusion in informed consent forms and discussion with participants. Medication tapers are frequently required prior to entry into psychedelic clinical trials, so rationale for these and their risks should also be covered, with a particular focus on how any emergent suicidality will be managed.

### Identifying a study drug supplier

For researchers conducting investigator-initiated studies, identifying a study drug supplier can be a challenge due to the small number of DEA-registered manufacturers of Schedule I psychedelics in the United States. In some cases, it may be necessary to import study drug, which necessitates working with an ex-US manufacturer/exporter, a DEA-registered importer, and DEA-registered distributor. The United Kingdom and Canada are important sources of Schedule I psychedelic study drugs.

To submit an IND to the FDA, the investigator will need a clinical protocol, investigator brochure, and chemistry manufacturing and controls (CMC) information (see below for dedicated section on the process of submitting an IND application). The ability to complete this information will depend greatly on the history of clinical research with the study drug that the FDA/European Medical Agency (EMA) certified Current Good Manufacturing Practice (cGMP) manufacturing company has supplied, as well as outside sources of information on that specific drug substance or drug product. As such, the selection of a drug supplier can greatly impact the feasibility, workload, and speed of an IND submission. Some suppliers may require payment before supplying documents to support an IND submission. If speed and workload are of primary concern, we recommend selecting a supplier who has a Drug Master File (DMF) on record with the FDA and is willing to provide a letter of authorization to access this information. This would in turn reduce the content needed for the CMC section of the IND. That is by far the easiest pathway to initiate a study. Likewise, selecting a supplier with a successful record of supporting IND submissions with the study drug ensures an easier path to approval. However, if concerns such as project funding, cultural fit between company and medical center, timing of providing study drug, amount of drug available, need for bulk vs. encapsulated drug, or shared population/indication are of equal or greater concern, we recommend careful review of the documentation that is provided with the drug. This can include the certificate of analysis for purity, short and long-term stability data of the drug substance or drug product and published or referenced material and data to be included in the investigator brochure. If it is necessary to import the study drug, once the IND is approved and the study drug code is officially on the investigator’s DEA Schedule I researcher registration, a DEA-registered importer can begin the application for a DEA import permit (DEA Form 357). The import/export permitting process is managed by the DEA Diversion Control Division Import/Export Section. They will coordinate all permitting and International Narcotic Control Board approval in a parallel and transparent matter. This process usually takes about 30 days to complete, so it is important to consider this when planning the study timeline. For industry sponsored studies, a sponsor’s drug supply chain management will assist in this process, which will entail close coordination with the foreign supplier/manufacturer, shipper, customs warehouse, and the DEA-registered importer and distributor. For investigator-initiated studies, the importer cannot be the researcher (unless you are conducting a Phase 1 study as a “coincident activity”), and it is unlikely that an academic medical center would ever become a registered importer of Schedule I drugs, so you will likely need to work with an import depot, which typically also has DEA manufacturing and distribution registrations.

Additionally, study drug manufacturing may differ between companies. For example, across the currently available psilocybin manufacturers, some provide the pure drug substance (powder in an amber vial), and others provide the drug product (encapsulated) at varying doses (e.g., 5 mg capsules, 25 mg capsules). If you plan to have an active placebo in your study (e.g., 1 mg psilocybin), many of the manufacturing companies also offer this option.

Any formulating, packaging, and relabeling effectuated after manufacturing of the bulk study drug can only be done by a DEA-registered manufacturer unless this is a “coincident activity” in a Phase 1 human trial (Milgram et al. 2022a). Importantly, this requirement does not apply to encapsulating powdered study drug or preparing intramuscular/intravenous preparations, though plans to do this should be clearly spelled out in your protocol. If necessary (an unlikely scenario for most researchers), obtaining a DEA Schedule I manufacturer registration requires cGMP compliance and an inspection by the FDA and DEA of relevant technology and facilities (i.e., compounding hood, clean room, etc.), followed by publication in the Federal Register of pending registration approval with a 60-day public comment period, a process which can take up to a year to complete.

### Submitting an IND to the FDA

There are two basic forms of an IND: IITs and sponsored applications, with specific implications for each. IITs often encompass early-stage feasibility, tolerability, or safety trials typically having a limited study population and scope. They may include initial, but likely underpowered, secondary outcomes of efficacy, whereas sponsored trials could include dozens of sites and many thousands of participants. IITs often have a somewhat lower bar to initiate given the typical exploratory nature and limited scope compared to sponsored trials. Sponsored trials typically are intended to support commercial product development and include substantial backend regulatory and other support from the sponsor. Both will require submission of an introductory statement and general investigatory plan, the clinical protocol, investigator brochure, CMC information, and pharmacology and toxicology data to proceed, which highlights the importance of the drug supplier for IITs. A full list of IND section requirements can be found in Table 2. Some drug suppliers do not offer their own investigator brochure to be included in an IIT’s IND submission, which puts the responsibility of producing one on the investigator. Although Investigator INDs can be submitted either electronically through the CDER NextGen portal or physically by mail, sponsor INDs must be submitted via the portal. Information on where to submit an IND can be accessed here: https://www.fda.gov/drugs/investigational-new-drug-ind-application/information-sponsor-investigators-submitting-investigational-new-drug-applications-inds.

**Table 2:** List of IND application contents

FDA Form 1571: Investigational New Drug Application (21 CFR 312.23(a)(1))
Table of Contents (21 CFR 312.23(a)(2))
Introductory statement (21 CFR 312.23(a)(3))
General Investigational Plan (21 CFR 312.23(a)(3))
Investigator’s Brochure (21 CFR 312.23(a)(5))
Clinical Protocol (21 CFR 312.23(a)(6))
- Study Protocol (21 CFR 312.23(a)(6))
- Investigator data (21 CFR 312.23(a)(6)(iii)(b)) OR completed Form FDA 1572
- Facilities data (21 CFR 312.23(a)(6)(iii)(b)) OR completed Form FDA 1572
- Institutional Review Board data (21 CFR 312.23(a)(6)(iii)(b)) OR completed Form FDA 1572
Chemistry, manufacturing, and control data (21 CFR 312.23(a)(7)) OR Letter of Authorization
Pharmacology and Toxicology Data (21 CFR 312.23(a)(8))
Previous human experience (21 CFR 312.23(a)(9))
Additional Information (21 CFR 312.23(a)(10))

After an IND application is submitted, the FDA will issue an acknowledgement letter, which includes the official date of receipt (United States Food and Drug Administration 2015). The FDA then has 30 calendar days to make a decision on the clinical protocol under the IND. The FDA will either allow the study to proceed or issue a clinical hold, which can be partial or complete. Partial holds may allow some trial activities for enrolled participants to continue while preventing new enrollments for a newly initiated or on-going study. Complete holds are usually issued in the case of new INDs and prevent a study from commencing in any form. To avoid issuing a clinical hold, the FDA will often contact the sponsor with preliminary comments during the review period. These comments can be delivered to the sponsor in multiple documents prepared by a multi-disciplinary team of reviewers and can arrive at any point within the 30-day review period. Importantly, they can sometimes arrive near the end of this period, leaving as little as 24–48 h to for the sponsor to respond to prevent the issuing of a clinical hold. Therefore, from a practical standpoint, investigators should not submit an IND during a period where they may not be able to address preliminary comments quickly to avoid a clinical hold. If the sponsor’s responses to the FDA’s preliminary comments are considered satisfactory, the FDA will then issue a “Study May Proceed” letter.

If the FDA does not issue a clinical hold within 30 calendar days of receiving an IND, this is generally considered implicit approval to begin study enrollment. The FDA may also issue a “Study May Proceed” letter to the sponsor prior to the end of the 30-day review period, allowing enrollment to begin sooner.

If a clinical hold is placed, it can be communicated via phone or writing. The sponsor will then receive a detailed written explanation within 30 days that lists deficiencies identified by the FDA and recommended approaches to mitigate them. Once the sponsor has submitted a response addressing the FDA’s concerns, the agency will respond within 30 calendar days about lifting the hold (U.S. Department of Health and Human Services 2020). If the sponsor’s response is satisfactory, the FDA will then communicate that the hold is lifted, which will be followed by a “Study May Proceed” letter. If a clinical hold is unresolved for over a year, the IND may become inactive (National Institutes of Health undated).

Detailed IND guidance information from the FDA can be found here: https://www.fda.gov/drugs/types-applications/investigational-new-drug-ind-application.

### Applying for a Schedule I researcher registration

To obtain a Schedule I researcher registration, applicants must submit a DEA Form 225 via the DEA’s web portal [Registrant Information Consolidated System (RICS)] along with (where applicable) the attachments listed in Table 3. Here are some important details regarding required attachments:

**Table 3:** List of DEA Schedule I researcher registration application attachments

Cover letter
Applicant’s curriculum vitae
Applicant’s state professional license
Applicant’s state-controlled substances license (not all states require this)
Applicant’s state drug distribution license (not all states require this)
Applicant’s DEA registration(s), to include prior Researcher and Practitioner Registrations
Signed study protocol
Site security agreement and standard operating procedure for study drug management
IRB approval letter for the study
Institutional approval letter (i.e., letter from a department chair or other administrator acknowledging that the study is permitted and “sufficient” to conduct)
FDA open IND acknowledgement letter
Drug distributor’s DEA registration with the appropriate Schedule (I)
Drug manufacturer’s DEA registration with the appropriate Schedule (I)
Drug importer’s DEA registration with the appropriate Schedule (I)
Drug distributor’s state registration (if applicable)
Drug export license (Foreign Government)
Controlled substance reverse distributor information

- Cover letter, which should include:
  - Study location.
  - Summary of the attachments.
  - Amount of study drug to be received. Since study drug can be packaged in different ways (i.e. bottles, blister packs, nasal spray devices, etc.), we suggest including the number of containers of study drug that will be received, the contents of the containers (for randomized controlled trials, you will need to state that the containers contain active drug product or placebo and for dose finding trials you will need to state that dose will vary and give the range), and the maximal amount of study drug the site can administer over the course of the study.
- Site security agreement should state security provisions for storing and dispensing the controlled substances to prevent diversion.
- Controlled substance reverse distributor information should either be a letter from distributor stating they offer the option for sites to return unused study drug for disposal or details of your arrangement with an approved DEA-registered Reverse Distributor.

Of note, the DEA website requires attachments to be in Portable Document Format (PDF). Only some attachments are explicitly requested by RICS. For others, we recommend uploading them as “Protocol” document type, which will allow you to upload multiple documents. Be sure to specifically label these documents so their purpose is clear from their title, since the document type for all of them will be “Protocol.” Uploading documents behind the “Protocol” upload button will attach the Project Name to all the additional documents so that DEA headquarters can easily distinguish the new submission package.

Once submitted, the Schedule I registration application is reviewed, and a local Diversion Investigator will be assigned to perform a site inspection to ensure compliance with drug inventory accountability, storage, and access control requirements. It is important to emphasize that there can be considerable differences in interpretation of these requirements between Diversion Investigators. Of note, none of the documents uploaded into the RICS can be accessed or viewed by the local DEA Diversion Investigator. Therefore, most Diversion Investigators will ask that you email them some of these documents after DEA headquarters has completed their review and approval.

You must, and your pharmacy should, be present for the site visit. In some states where a state level inspection is also required, the federal and state inspections may be conducted simultaneously. In some cases, where you have a current Schedule I researcher registration, the on-site visit may be waived in lieu of photos of any secure storage container to be used for the new study drug.

The DEA will require that the state license be issued **prior** to the DEA approving the application for a new submission or adding a new drug code to an existing Schedule I researcher registration if state licensing is required. We are aware of at least one state-controlled substance board (Massachusetts) that asks for a DEA Schedule I license number in its application for a state Schedule I license. If this occurs, we recommend discussing how to proceed with the state-controlled substance board and your Diversion Investigator. Some states will work in parallel with the DEA, but this is an exception to the rule. Some states (Connecticut, Georgia, Maryland, South Carolina, and Wisconsin) require a DEA-registered distributor or manufacturer to be licensed in that specific state as a Non-Resident Pharmacy. These non-resident pharmacy requirements involve a board or commission approving such activity and could add several months to your timeline.

Required DEA documentation to be maintained throughout the study includes:

- Drug accountability logs that document study drug administrations, which participant received study drug, and who administered it.
- Delegation of authority log, which details study staff responsibilities.
- Drug storage entry/access logs, which are updated every time study drug is accessed.
- Written authorization for any practitioner or non-practitioner to be a secondary dispenser of the study drug under the authority of the Schedule I license holder. The name, address, date of birth, and social security number of secondary dispensers should be provided to your Diversion Investigator. Schedule I researcher registration holder and secondary dispensers must have a limited background investigation that asks the required questions in 21 CFR 1301.90 (United States Code of Federal Regulations 2024f). A study sponsor may conduct background checks of clinical staff who have access to and/or handle Schedule I study drugs.

Once the Schedule I application is approved, the registrant must request their pre-printed DEA Form 222s from the DEA Diversion Control Division Registration Section. Study drug is ordered from the DEA-registered distributor using the DEA Form 222. Only the DEA-registered researcher acting as the principal investigator or their designated power of attorney can sign a DEA Form 222 to order the study drug from the supplier and sign for the receipt of a Schedule I study drug that is specific to the DEA-registered researcher’s drug code. This can create challenges since delivery services usually make deliveries to hospital receiving divisions with little notice. Therefore, study staff should track shipments of study drug online and maintain close communication with receiving division staff to ensure an authorized study staff member signs for receipt.

Any amended or supplemental protocol must be reported to the DEA throughout the course of the study via RICS. An Amendment, in DEA terms, means a change to the amount of study drug used or change in formulation. Supplementals are changes to the study parameters such as new screening thresholds that do not change the drug dosing amounts. Both must be reported, and although DEA Research Manual says that you may not proceed until DEA provides specific approval to proceed, the DEA will most often not respond with any communication on supplemental protocols. Therefore, if you require confirmation that the supplemental protocol has been reviewed, you may need to contact your Diversion Investigator. Your Diversion Investigator may conduct announced or unannounced visits to ensure compliance. We are aware of at least one site that received an unannounced DEA visit coincidentally during a treatment session. Though this visit was brief and had no impact on the study protocol or participant’s experience, study staff should nevertheless be prepared to manage this contingency. We strongly recommend reviewing the DEA Researcher’s Manual for further information related to Schedule I researcher registration requirements (Milgram et al. 2022b) and forming a strong working relationship with your Diversion Investigator.

### Adding additional Schedule I drugs to your Schedule I researcher registration

Adding additional Schedule I drugs to an existing Schedule I researcher registration requires the same documentation as if you are submitting a new application for a DEA Schedule I researcher registration, though it is performed via a registration update rather than a new license application. This involves selecting the new drug code and uploading the new study’s protocol, as well as other relevant documents. While applications should expect the DEA’s response time for this to be the same as that of a new application submission, some investigators report a faster turnaround time. Certain requirements, such as an on-site inspection may be waived depending on your Diversion Investigator. Importantly, you may be assigned different Diversion Investigators for different Schedule I drugs on your Schedule I researcher registration. When uploading relevant documents for the new Schedule I drug, be sure to label them clearly with the name of the new drug since documents associated with the current drug and previously approved drug(s) are stored in the same researcher file in RICS.

Of note, we do not recommend submitting multiple applications to add Schedule I drugs to your Schedule I researcher registration concurrently. Due to limitations with the way documents are received via RICS, it is not possible for the DEA to approve concurrent applications individually. Therefore, if there is a delay in the approval of one drug application, you will not be able to secure approval for the other drug application if they were submitted concurrently.

### Psychedelic treatment session logistics

Given the long-lasting psychoactive effects of many psychedelics, plan for treatment session days in advance to ensure adequate staffing. Having clear communication among team members about time off well in advance of scheduling treatment days is vital to avoiding protocol deviations. Clinicians should also ensure there is coverage for their typical inpatient or outpatient duties, including phone calls and EMR messages from patients about urgent issues. Due to the need for participants to always be accompanied by at least one monitor, it is advisable for an additional monitor to be available to cover breaks for the primary monitor or monitors. A study staff member may also need to be available to process or ship laboratory samples drawn on treatment session mornings. A physician is usually required to complete the treatment session discharge checklist for participants, which can create scheduling challenges. While a guiding principle of most psychedelic treatment protocols is to provide support and assurance while also assisting the participant in processing any potential psychological challenges, a back-up plan should be in place with rescue medications including anxiolytics and second generation antipsychotics (SGAs) with 5HT-2 A antagonism [such as risperidone or olanzapine (Valeriani et al. 2015)] as part of the study protocol. These can be used for distress or behavioral dysregulation refractory to reassurance. A recent clinical trial evaluating lysergic acid diethylamide (LSD) involved the administration of lorazepam and olanzapine to a participant for anxiety and delusions (Psychedelic Alpha 2022). Of note, first generation antipsychotics (FGAs) such as haloperidol lack the 5HT-2 A antagonism of SGAs [psychedelics primarily exert their psychoactive effects via 5HT-2 A partial agonism (Kometer et al. 2013; Becker et al. 2023)] and may exacerbate psychedelics’ psychotomimetic effects (Vollenweider et al. 1998). Furthermore, benzodiazepines might attenuate the antidepressant effects of psychedelics (Hibicke et al. 2024).

For participants who will undergo neuroimaging while experiencing psychedelic effects, a clear plan should be developed for safe transportation to and from the treatment area to the neuroimaging scanner. In addition, since neuroimaging procedures may be particularly likely to elicit aversive reactions to psychedelics (Studerus et al. 2012), researchers should give special consideration to participant selection and preparation, including informed consent, and consider how to best ensure participant comfort and safety in these circumstances. Sites should also have a standard operating procedure prepared for evacuating a participant to a safe location in the event of an emergency, such as a fire, during a treatment session. Sites should also have a plan for contacting security if a participant tries to elope, as well as transfer to an emergency department or inpatient psychiatric facility if a participant develops suicidality, agitation or psychosis that cannot be controlled with de-escalation interventions or rescue medications. If planning to conduct neuroimaging, we recommend allowing participants to familiarize themselves with the scanner and imaging suite (and any additional imaging personnel) in advance of their first treatment session.

### Psychedelic clinical trial recruitment challenges

Recruitment challenges may occur due to a variety of issues with potential participants, including stigma specific to psychedelics (and opposition to psychoactive drugs and pharmacotherapy more generally), substance use history, limited number of participants meeting diagnostic criteria for the target condition, inability of participants to safely wean off other psychoactive drugs, and interest from volunteers seeking treatment for conditions outside the scope of the study. Due to growing public interest in psychedelics (Danias and Appel 2023) and associated media hype (Yaden et al. 2022), psychedelic clinical trials can face expectancy bias from potential participants. Media coverage related to psychedelics has long been believed to pose challenges for conducting psychedelic research, with extensive media coverage of potential adverse effects of LSD in the late 1960s possibly leading to decreased interest in participation in LSD trials and heightened anxiety in study participants (Dahlberg et al. 1968). Excessive interest among higher-resourced populations (Bogenschutz et al. 2022) and limited interest from groups with higher perceived risk of psychedelics (Barnett et al. 2024) and/or a history of exploitation within medicine (e.g. African Americans and Indigenous peoples) (Thrul and Garcia-Romeu 2021; Ortiz et al. 2022) also present important potential limitations to generalizability of findings.

If study staff participate in media interviews about their psychedelic research, this can be a particular problem in the weeks afterward, with study coordinators becoming overwhelmed by a deluge of interest, depending on study location and the condition being investigated. Unfortunately, this interest often comes from people seeking treatment for conditions that are not being investigated in the trial or people who have used psychedelics repeatedly or recently (which is disqualifying for most psychedelic clinical trials), yielding few participants who meet study inclusion/exclusion criteria. Establishing an IRB-approved screening page (e.g. through electronic data capture (EDC) systems like REDCap (Harris et al. 2009) that potential participants can fill out on their own without the involvement of a study coordinator can be an effective tool to reduce administrative burden.

Another possible area of difficulty is the interest of potential participants, usually of means, who live far from the study site. While it may be tempting to enroll these participants, recruitment needs must be balanced against the risk of loss to follow up, particularly if the individual receives placebo, as well as the potential difficulties of managing adverse events from a distance.

### Managing psychedelic clinical trial participant disappointment

Because of media hype, investigators should expect to manage high levels of disappointment in some potential participants who screen fail, participants who receive placebo (and recognize it), and participants who receive a psychedelic but have a suboptimal response. Sometimes the disappointment of potential participants who are disqualified from study entry during pre-screening phone calls or during screening visits may lead them to escalate complaints that they have been unfairly excluded to the study sponsor or institutional authorities. Studies employing a placebo control bear special mention. Many colleagues in the field have recounted that no matter how many times study staff explain that there is a high chance (often 50%) of receiving placebo, participants may be extremely disappointed when this occurs, in some cases leading to drop out. To counter this risk and potentially reduce the effect of expectancy, some trials utilize an open-label crossover design, offering active study drug to all participants or participants who received placebo after primary endpoint data are collected (Bogenschutz et al. 2022).

### Considerations for non-psychedelic treatment session study visits

Due to increasing reliance on central raters blinded to treatment allocation and visit number in industry sponsored clinical trials [in line with FDA recommendations (United States Department of Health and Human Services 2023)], space should be available for participants to participate in these calls or video evaluations at follow up visits. Participants may need to use electronic tablets for these visits due to confidentiality issues preventing them from using staff computers while unattended. If this is the case, Wi-Fi access will be necessary, and cybersecurity will likely need to approve tablet use.

### Considerations in psychedelic preclinical (non-human) research

Many of the same challenges are present for conducting preclinical research with psychedelics as with clinical research. However, one does not need approval from the FDA for the former, and such issues related to IND submissions are not present. However, psychedelics remain a Schedule I drug regardless of the research purpose. As such, a Schedule I researcher registration must be obtained from the DEA and all the same procedures for storing drugs, monitoring drug administration, disposing drugs, and preventing diversion must be followed. Rather than gaining approval of the clinical protocol from the IRB, approval of the research protocol must be obtained from the Institutional Animal Care and Use Committee (IACUC) if it involves vertebrate animals. The IACUC is composed of an institutional, veterinary, community, scientific, and bioethics membership at minimum, but they vary in size, speed, complexity, culture, and process. The operation of the IACUC is governed by the Animal Welfare Act and both minimum and best practices are outlined in the Guide for the Care and Use of Laboratory Animals (Institute for Laboratory Animal Research 2011). All research will also be subject to biosafety approval and possibly to approval by the radiation safety office. Special treatment of animal and tissue specimens exposed to Schedule I substances per DEA guidelines is discussed below.

### Disposal of study drug and specimens exposed to study drug

In addition to careful record-keeping on storage and usage of the controlled substance, the DEA requires strict procedures for disposal of any unused Schedule I study drug. At the time of Schedule I researcher registration application submission, you should provide detailed information on how you plan to destroy unused drug. Your institution’s pharmacy may have established approved methods of destroying controlled substances or may have a contractual relationship with a DEA-registered Reverse Distributor. Applicants planning to conduct animal or bench research should be aware that animals, tissues, or organoids exposed to a Schedule I substance may also need to be destroyed according to DEA rules and regulations, which may add additional layers of complexity and the potential need for additional external services. Details on disposal, as well as many other aspects of the license holder’s responsibilities, including how to handle study drug theft or loss, can be found online in the DEA Researcher’s Manual (Milgram et al. 2022b).

Blinded and unblinded unused study drug is an area that the DEA Pharmacist’s Manual (Milgram et al. 2022c) and the CFR (United States Code of Federal Regulations 2024 g) address, though not comprehensively, so it is recommended that you consult with your Diversion Investigator before transferring unused blinded study drug to a sponsor’s depot or a reverse distributor. Part 4 of the DEA Form 222, which is used to order and transfer Schedule I-II controlled substances, requires that a specific drug code be inserted by a “supplier.” When sending unused Schedule I study drug back to the depot that supplied you, or transferring to a Reverse Distributor, you will be the “supplier” for the purpose of the DEA Form 222. If you are blinded, it is not possible to fill out Part 4 of DEA Form 222. You will have to closely coordinate this type of transfer with a sponsor, the sponsor’s depot, and your Diversion Investigator.

### Unusual situations arising in psychedelic research

Terrance McKenna alleged that Timothy Leary once quipped, “LSD occasionally causes psychotic behavior in people who have not taken it (McKenna 1993),” a statement which can provide helpful context for the unusual situations that sometimes arise around psychedelic research. Based on our experiences, the mere act of conducting research with psychedelics can elicit strange, sometimes problematic behavior, in people within one’s institution or the community who have limited or no study involvement. Here we list illustrative examples from our own experiences:

- The review of a contract for an industry-sponsored psychedelic clinical trial was delayed after an attorney in the legal department of the academic institution planning to serve as a trial site became incorrectly convinced that the trial involved dosing unwilling participants with a psychedelic. This was even though the trial had an FDA-approved protocol and thorough informed consent form, which the attorney was being asked to review.
- An IRB requested to evaluate the music playlist of a psilocybin clinical trial, indicating that whereas classical music would be acceptable, music of the psychedelic genre and specifically of the American improvisational rock band Grateful Dead would raise concern. It is unclear whether content of music playlists falls under IRB purview, and if it did, how genres or bands that lack explicit content and have enjoyed mainstream success might violate ethical or safety standards. The investigators interpreted the IRB comments as an expression of its unease around the association of psychedelics with the countercultural revolution of the late 1960s.
- An undergraduate on campus reportedly selling “psilocybin” falsely claimed the drug was from the surplus supply of an ongoing psilocybin clinical trial at the university (presumably to increase the sales appeal of his product). After hearing about this situation, the investigator reported it to the DEA out of an abundance of caution.
- Two patients admitted to a hospital’s psychiatric facility with psychosis falsely claimed they had been participants in a psychedelic clinical trial at that hospital.
- Nurses who overheard that a psychedelic trial might be happening on their floor escalated their concerns about participant safety to department administration.
- During screening, several potential participants reported recent exposure to media coverage of psychedelics and indicated the willingness to participate only if randomization to psilocybin (as opposed to the placebo) could be guaranteed. Upon being apprised of random assignment procedures, some of these potential participants expressed the intention to engage in naturalistic psychedelic use if they were randomized to receive placebo. None of these individuals were enrolled.

With these episodes in mind, we hope new researchers in psychedelic medicine will not be caught off guard if they experience similar situations. Due to lingering stigma and likely unconscious discomfort with the potent psychoactive effects known to be unleashed by psychedelics, we unfortunately must also encourage aspiring researchers in this space to be prepared for raised eyebrows and innuendo-laden comments, even in the most serious of research environments.

## Conclusion

Compared to investigations of existing behavioral and pharmacological interventions for mental health conditions, conducting research with psychedelics is fraught with significantly greater challenges. Investigators intent on establishing psychedelic research programs should expect to encounter multiple implementation barriers including securing institutional administrative support, acquiring funding, obtaining approval for an IND application from the FDA, clinical grade drug sourcing, obtaining a Schedule I researcher registration from the DEA and an equivalent state license (if required), preparing spaces for treatment and study drug storage, implementing DEA and in-house rules for controlled substance storage and management, and engaging the local community. However, these obstacles are likely surmountable for investigators in most academic medical centers with significant planning, persistence, and efforts to build institutional support early on.
